# Ptotic Gall Bladder with Hepatic Masses: A Case Report

**DOI:** 10.1155/2013/854686

**Published:** 2013-02-11

**Authors:** Hasan Aydin, Z. Banu Aydin, Baki Hekimoğlu, Ayşe Görmeli

**Affiliations:** Radiology Department, Diskapi Yildirim Beyazit Education and Research Hospital, 06660 Ankara, Turkey

## Abstract

Gall bladder (GB) may be found in a variety of abnormal positions. Most of them are due to arrested development of embryonic growth at different stages. A 63-year-old female patient was admitted to our radiology unit for magnetic resonance imaging (MRI) of the liver for the lesions identified in abdominal ultrasonography (US) and computed tomography (CT). MRI showed that there was a lobulated heterogenous mass in the left lobe of the liver and a smaller one in the right lobe of the liver with the same appearance. The inferior pole of the liver was located in the pelvic space, and the GB, which contained sludges and stones, was lying down to the upper pelvic space. Hepatic masses were considered to be hemangiomas, and GB was diagnosed as ptotic GB with luminal sludge and stones. In this case, especially, MR imaging helped the surgeon to plan a proper approach to the GB in abnormal localization.

## 1. Introduction

 GB is located adjacent to the undersurface of the liver at the junction between left and right lobes. It is mostly found in the right upper quadrant but may be seen in any part of the abdomen [1]. Although anomalous positions of the GB are rare, the most common aberrant locations are under the left hepatic lobe, intrahepatic, transverse, retrohepatic, retroduodenal, and retroperitoneal positions, and at the same time, it has also been reported in lesser omentum, falciform ligament, within muscles of abdominal wall, and within the thorax [2, 3]. 

To our knowledge, ptosis of the GB is a rare very important entity, as it can create a serious clinical confusion. 

If the location of the GB is away from the peritoneum or retroperitoneal, the typical clinical signs of cholecystitis can be absent, and symptoms of peritoneal irritation may not be seen. A GB lying outside its normal anatomic fossa may be susceptible to torsion and consequent gangrene [4].

If the routine preoperative studies may not detect the aberrant location, an unusual surprise for the surgeons during laparoscopy may occur precedingly [5].

We report here a case with ptotic GB and liver masses. The US, CT, and MRI findings of the patient will be presented.

## 2. Case Report

A 63-year-old woman was admitted to the hospital with lower quadrant pain. On physical examination, there was hepatomegaly with soft and mildly tender abdomen in the right lower quadrant without peritoneal signs. There was no Murphy's sign on examination.

**Figure 1:**
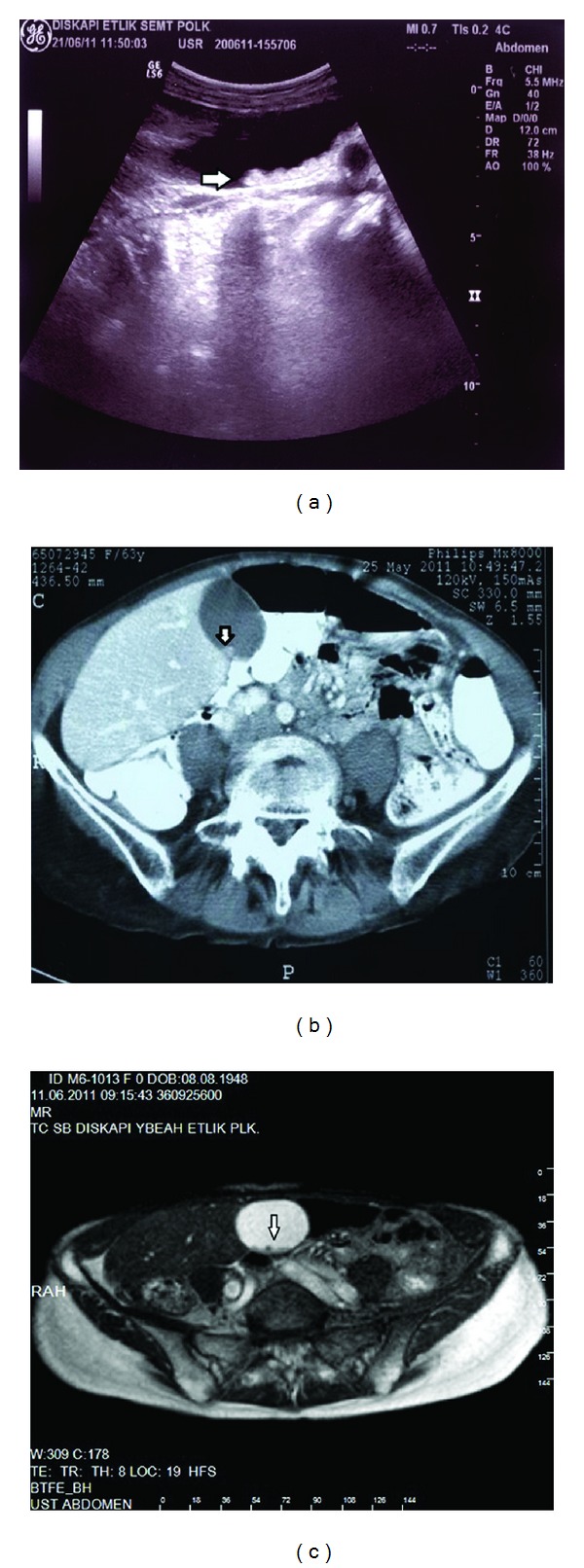
There was intraluminal sludge and gallstones of the GB (arrows). ((a) US, (b) CT, and (c) MRI findings).

**Figure 2:**
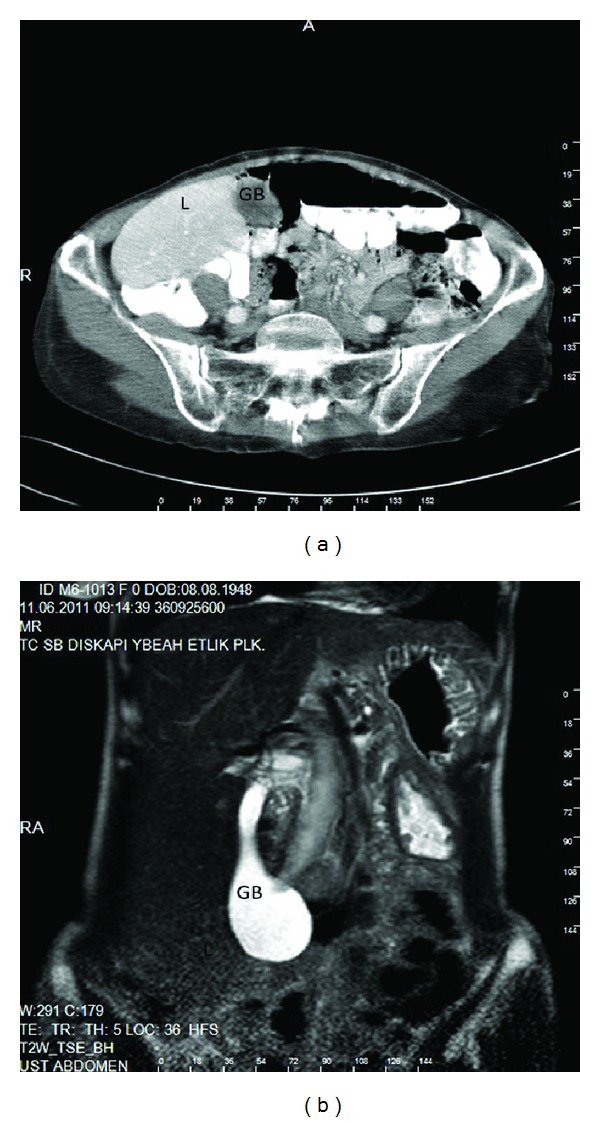
The GB was located in the right lower quadrant (ptosis). ((a) CT and (b) MRI findings) L: liver and GB: gallbladder.

**Figure 3:**
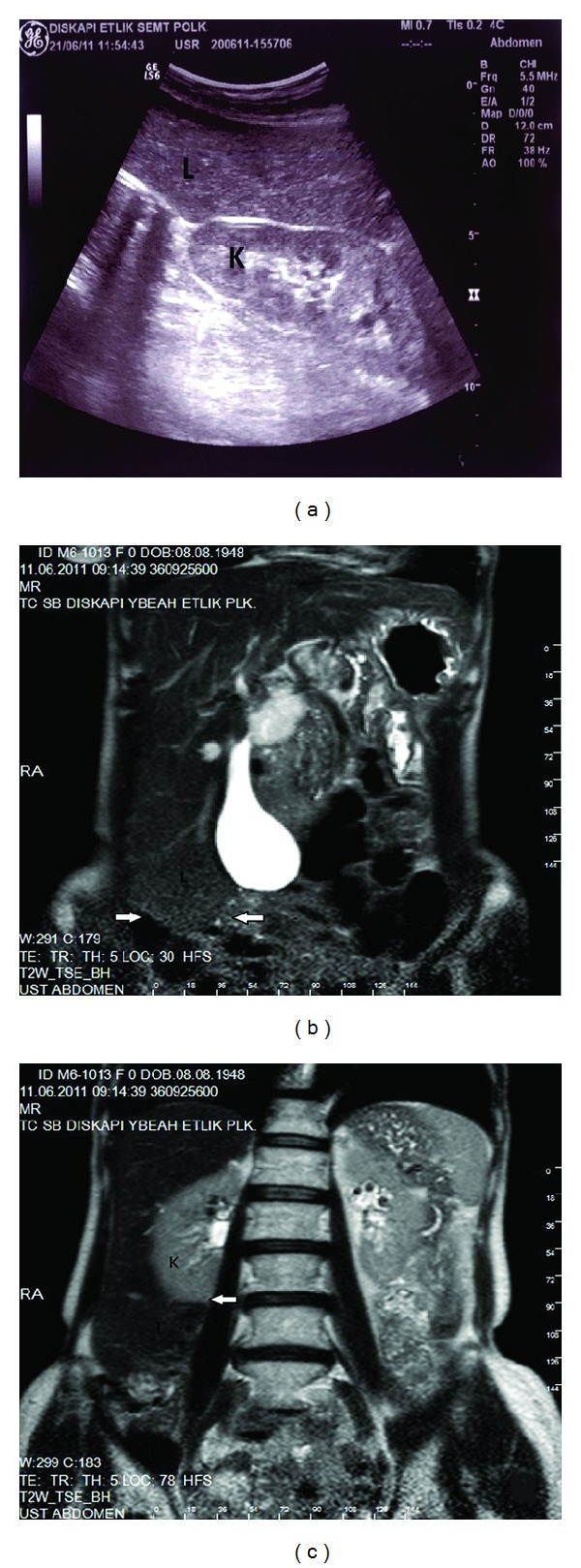
(a) There was hepatomegaly on US imaging. (b) Coronal plane of MRI showed that the inferior pole of the liver was located in the pelvic space (arrows) and (c) the inferior pole of the right kidney was covered by the liver (arrow), L: liver and K: kidney.

**Figure 4:**
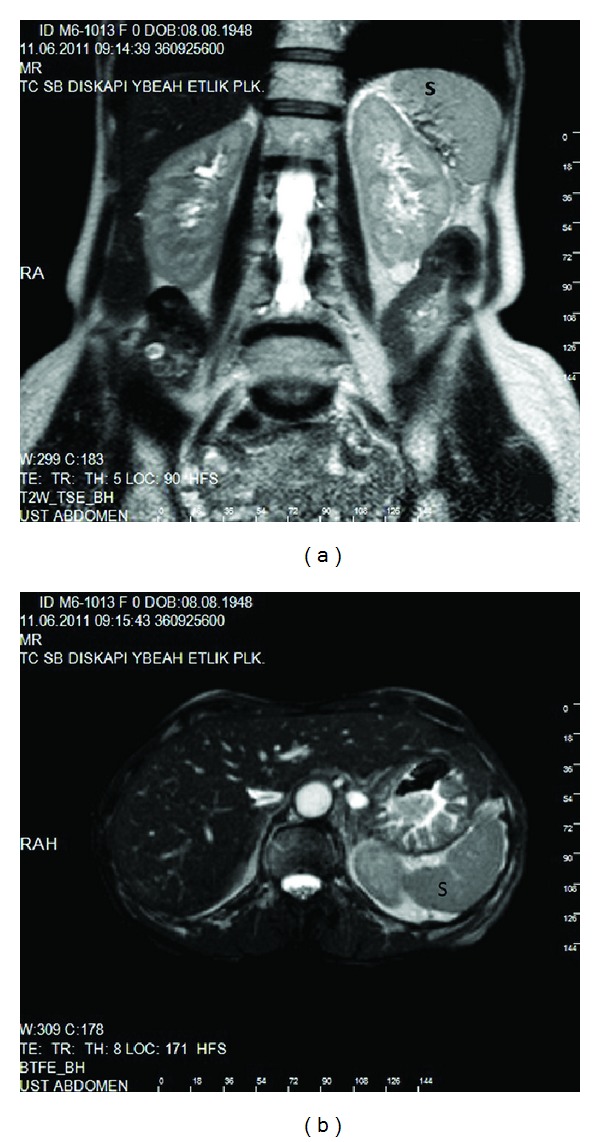
The spleen was atrophic on MRI ((a): axial and (b): coronal MRI) S: Spleen.

**Figure 5:**
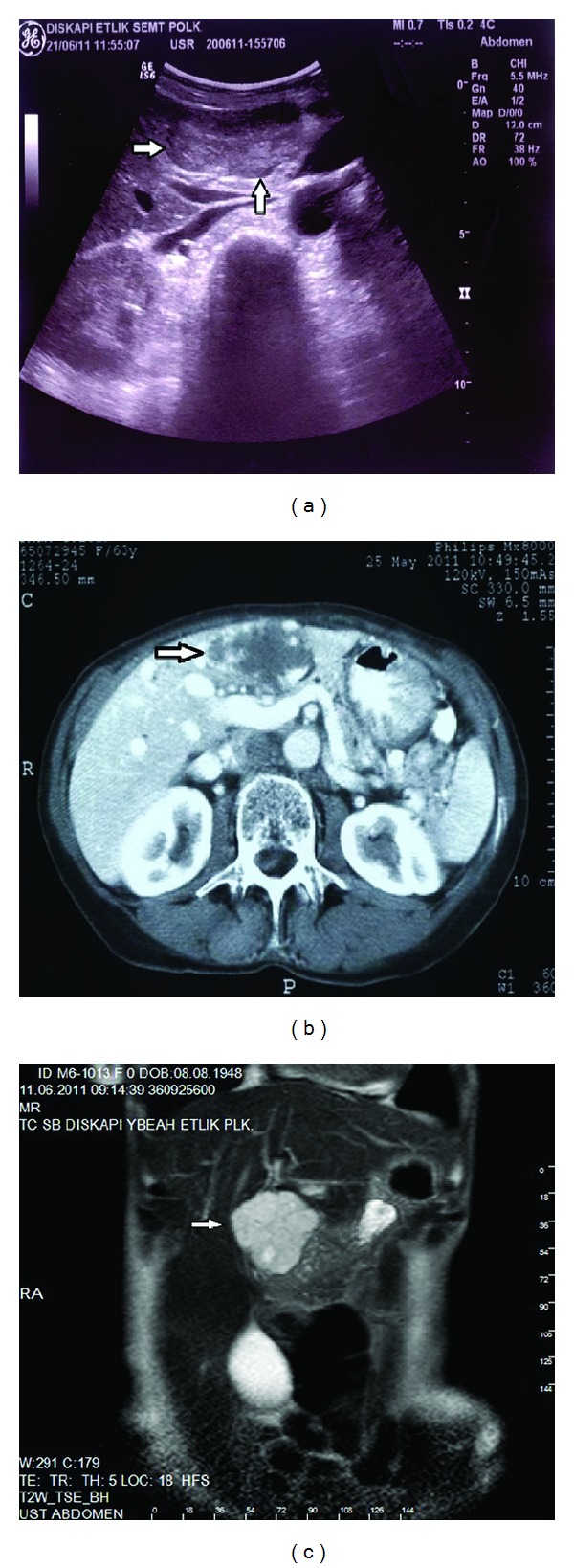
(a) A lobulated and countered, iso-hiperechogen liver mass in the left lobe on US imaging (arrows), (b) on postcontrast CT scan, nodular enhancement was seen at the periphery of the lesion (arrow). (c) On coronal T2W images, the lesion was hyperintense (arrow).

In the laboratory data, serum bilirubin was normal (total bilirubin: 0.6 mg/dL, direct bilirubin: 0.1 mg/dL), ALT and AST were normal with values of 23 IU/L and 17 IU/L, and alkaline phosphatase was normal (56 IU/L). Gama glutamyl transferase and lactate dehydrogenase were elevated with values of 76 IU/L and 255 IU/L. Serum electrolytes were normal (sodium: 138 mEq/L, potasium: 4.5 mEq/L, chloride: 103 mEq/L). On US examination, the GB with endoluminal sludges and stones was lying down to the upper pelvic space (ptotic GB) (Figure 1). The bladder wall thickness was normal. There was hepatomegaly (craniocaudal size of the right lobe of the liver was 225 mm), and the inferior pole of the liver was seen in the right iliac fossa next to the right adnexial region (Figure 2). The left lobe of the liver was also enlarged, and size of the spleen was 70 mm and seemed to be atrophic. There were cortical cystic lesions less than 10 mm in size, at the inferior pole of both kidneys. There was a lobulated, countered, iso-hyperechogeni, and heterogenous liver mass in the left lobe with 50 × 55 mm in size, seen in US (Figure 3), and a well-defined, homogenous, and hiperechogen liver mass measured 14 mm in the right lobe. CT was performed due to the evaluation of liver masses, and on CT imaging, the GB was ptotic as seen on US, and there were intraluminal sludge and gallstones (Figures 1 and 2). The spleen was atrophic (Figure 4), and there were cortical cysts at both kidneys. The liver was enlarged, and the heterogenous liver mass defined on US was hypoattenuating compared with adjacent hepatic parenchyma on unenhanced images. The other lesion defined in the right lobe could not be distinguished from the adjacent hepatic parenchyma on unenhanced images. After administration of intravenous contrast medium (Gadovist, 0.2 mL/kg), nodular enhancement was seen at the periphery of both lesions (Figure 5). 

**Figure 6:**
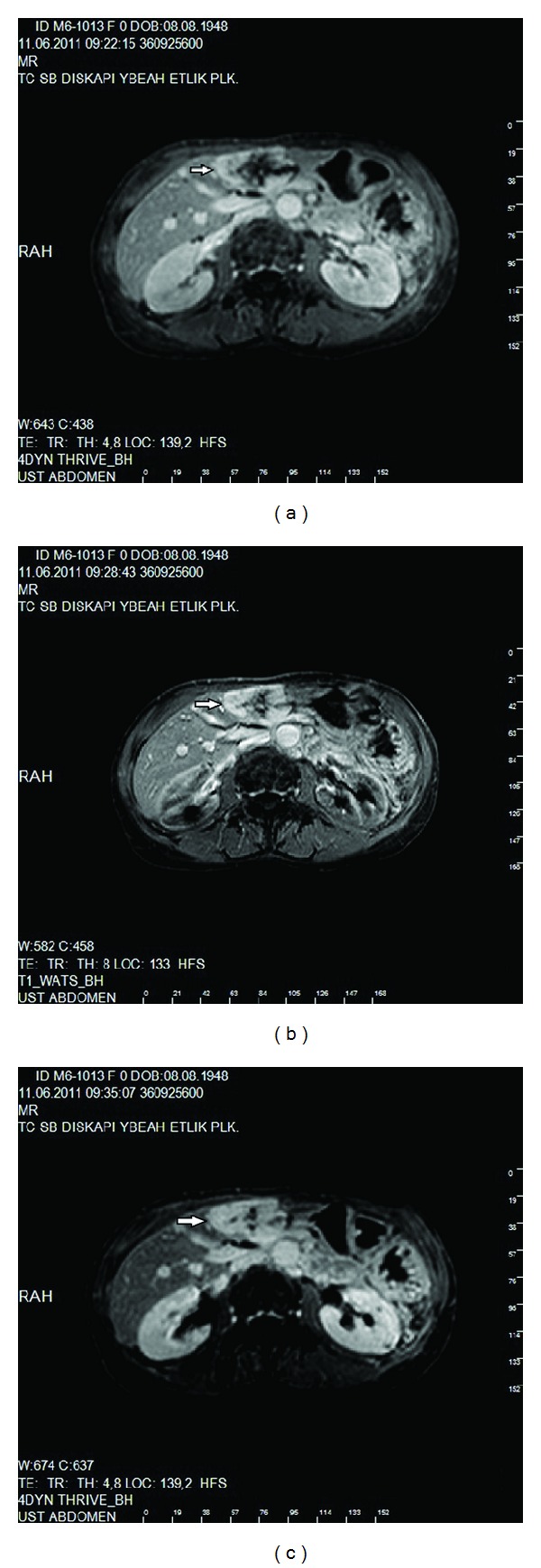
(a) On precontrast T1W images, a lobulated hyperintense liver mass in the left lobe with central-peripheral scar tissue (arrow). (b) On postcontrast early arterial MRI, the lesion showed hyperintense peripheral nodular enhancement (arrow). (c) On postcontrast delayed MRI, increasing central enhancement was seen in the lesion (arrow). The central-peripheral scar tissue did not show any enhancement on all images.

On MRI, dynamic series were performed to characterize the hepatic masses. The lesion in the left lobe predicted early peripheral nodular enhancement with the increasing central enhancement on delayed images. There was fibrotic scar tissue at the peripheral and in the centre of the lesion. The scar tissue did not enhance all images. With regard to all these findings, the lesion was considered to be a papillary hemangioma (Figure 6). 

**Figure 7:**
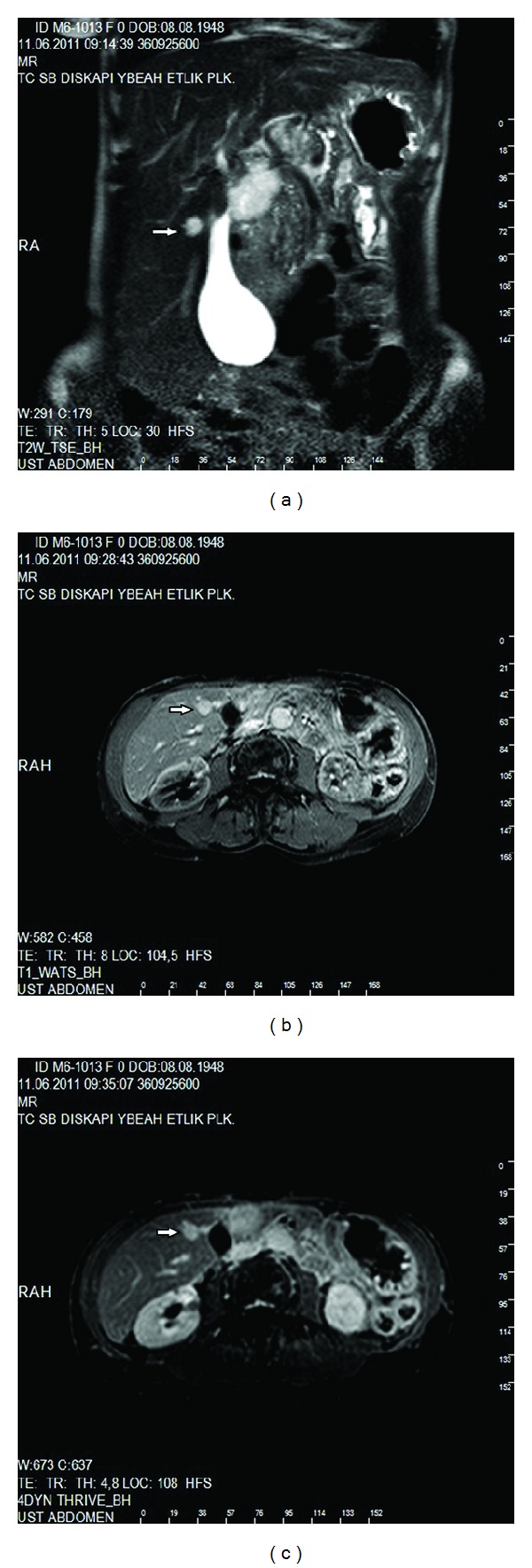
(a) On T2W images, a well-defined, homogenous, and hyperintense liver mass in the right lobe (arrow). On postcontrast T1W imaging, the lesion showed early hyperintense peripheral nodular enhancement (b) with increasing central enhancement on delayed images (c) (arrows).

The lesion in the right lobe presented the same enhancement pattern like the one in the left lobe except for the enhancement of central scar tissue and also was considered as a hemangioma (Figure 7). The ptotic GB, hepatomegaly, atrophic spleen, and the liver masses were confirmed with MRI, and in addition to these findings, the inferior pole of the right kidney was covered by the liver on coronal planes of MRI (Figures 1, 2, 3, and 4).

An operation for the GB and/or hepatic masses was not planned by the surgeon, but clinical followup in short intervals was recommended. On these circumstances, our radiologic findings were correlated with the clinical findings. 

## 3. Discussion 

An aberrant GB situated under the left lobe of liver, medial to the falciform ligament, was first described by Hochstetter in 1886 and was termed as “left -sided GB” [6]. It usually occurs as a component of situs inversus [5]. Transposition of only GB is seen up to 0.3% of the population [7]. The associated anomalies to the GB transposition include biliary system, portal vein anomalies and hepatic segment IV atrophies [8–10]. 

The GB rotation and/or displacement can be caused by hepatic lobe abnormalities, such as aplasia, hypoplasia, hypertrophy and by abnormal mobility of the GB itself [1].

Bender et al. [11] reported a left-sided GB. “During laparoscopic cholecystectomy, it was discovered that GB was at the left of the falciform ligament, with duplicated common bile duct. They noticed that as soon as left-sided GB was reported, a cholangiogram was performed in order to be a guide for the dissection because of the associated anomalies.” In our case, MRI was useful to describe the associated anomalies, but at the same time specific imaging like MRCP might be performed for an abnormality in the bile ducts as it was recommended in left-sided GB. 

Saygun et al. [12] reported a case about infracolonic malposition (under transverse colon) of the GB. “The fundus, corpus, and infundibulum of the GB were situated in the peritoneal layers of the transverse mesocolon, so a laparotomic approach with a right subcostal incision was performed in order to verify the occasion.” In our case, surgery was not performed and MRI results aid the surgeon to reach a proper diagnosis.

Reddy et al. [5] reported a case of laparoscopic cholecystectomy for left-sided GB. “The GB was severely diseased with lying to the left of the falciform ligament.” In our case, there were not any signs for severely diseased GB. The wall thickness and the size of the GB were normal, and there were no signs of peritoneal irritation.

Qureshi and Awad [13] and Meeker and Lisenby [14] reported an aberrant presentation of the GB during laparoscopic cholecystectomy. “An intraoperative cholangiogram was obtained to delineate the biliary anatomy that showed the cystic duct entering the common hepatic duct on the right side.”

Although these cases about left-sided GB were present in the literature, to the best of our knowledge, there were just two cases of ptotic GB. 

Meeker and Lisenby [14] reported a case of ptotic GB with a long pedicle with floating kidney in the abdomen. In our case, there were no other anomalous positions of abdominal organs except for GB.

Maki et al. [15] reported a GB ptosis and wandering GB. In the wandering of GB by contraction of the diaphragm, peristaltism of the colon, or change in posture, the GB may rise or decline by itself. In our case, the GB was in the lower quadrant, and its position did not change with breathing or with patient's position.

When a ptotic GB was discovered, the findings indicate that there might be associated anomalies such as ptosis of other abdominal visceral organs. Surgeons should be aware of these anomalies and should initiate the dissection as so close to the GB as possible [11]. The pedicle and neck of the gall bladder might be susceptible to the torsion and consequent gangrene. Cholecystectomy should be performed in ptotic gall bladders, even in asymptomatic cases, as it was recommended in ectopic gall bladders [16].

In our case, surgery was not performed, and clinical follow-up was recommended by the surgeon: that was the main limitation of this report.

Finally we believe that this is the first reported case of ptotic GB with hepatomegaly, liver masses, and atrophic spleen. Radiologic approaches especially MRI should be performed for detecting the associated anomalies and helping the clinicians to manage the proper approach for all patient's gall bladder problems [17, 18].
